# Healthcare services utilisation among patients with hypertension and diabetes in rural Ghana

**DOI:** 10.4102/phcfm.v12i1.2114

**Published:** 2020-07-30

**Authors:** Mercy N.A. Opare-Addo, Francis A. Osei, Kwame O. Buabeng, Afia F. Marfo, Isaac Nyanor, Evans X. Amuzu, Daniel Ansong, Ellis Owusu-Dabo

**Affiliations:** 1 Department of Pharmacy Practice, Faculty of Pharmacy and Pharmaceutical Sciences, College of Health Sciences, Kwame Nkrumah University of Science and Technology, Kumasi, Ghana; 2 Kumasi Centre for Collaborative Research in Tropical Medicine, College of Health Sciences Kwame, Nkrumah University of Science and Technology, Kumasi, Ghana; 3 Research and Development Unit, Komfo Anokye Teaching Hospital, Kumasi, Ghana; 4 School of Medical Sciences, College of Health Sciences, Kwame Nkrumah University of Science and Technology, Kumasi, Ghana; 5 School of Public Health, College of Health Sciences, Kwame Nkrumah University of Science and Technology, Kumasi, Ghana

**Keywords:** non-communicable diseases, hypertension, diabetes, rural, Ghana, healthcare preference, healthcare utilisation

## Abstract

**Background:**

Non-communicable diseases (NCDs) remain a global burden and is projected to increase due to aging, rapid urbanization and unhealthy lifestyles. The study was conducted to determine the prevalence of hypertension and diabetes in rural districts in the Ashanti region of Ghana and to determine factors that influence utilization of health care services.

**Setting:**

Offinso North, Amansie West, Ahafo Ano South, and Asante Akim South.

**Methods:**

A population based prospective cross-sectional study comprising of adults aged 18 years and above was carried out from January 2016 to March 2016. A multistage sampling method was employed to select four rural districts in the Ashanti region of Ghana. A structured questionnaire was used to collect primary data from respondents.

**Results:**

A total of 684 participants were recruited in the study. The prevalence of hypertension and diabetes was found to be 16.23% and 5.41% respectively. The prevalence of diabetes and hypertension comorbidity was 1.61%. The public hospital was the most preferred choice of facility (52.56%) for patients with NCDs.

Educational level significantly decreased the likelihood of seeking healthcare in OTCMS and Health Centre to Hospitals (RRR = 0.1, 95% CI = 0.011–0.917, *p* = 0.042) and (RRR = 0.4, 95% CI = 0.198-0.679, *p* = 0.001) respectively.

**Conclusion:**

The prevalence of self -reported hypertension and diabetes observed in this study was relatively lower for hypertension and higher for diabetes as compared to other studies in Ghana. The public hospital is the most preferred choice of health facilities for patients with hypertension and diabetes in the rural districts.

## Introduction

Non-communicable diseases (NCDs), type 2 diabetes and hypertension in particular have been found to be the leading cause of death worldwide. Sixty-three per cent of all annual deaths are presently attributed to NCDs, which amounts to over 36 million people each year. About 80% of these deaths occur in low- and middle-income countries.^1^ Non-communicable diseases are medical conditions that are not caused by infectious agents and are also referred to as chronic diseases that last for long periods of time and progress slowly. They are projected to account for more than a third (66%) of the global burden of disease by 2030.^2,3^ Non-communicable diseases are presently considered a global burden that constitutes a major public health threat, which impacts on the social and economic development worldwide.^1^ The World Health Organization (WHO) therefore advocates for efficient preventive strategies to halt the growth of NCDs.^4^ However, this growing burden does not seem to have been addressed over the years.

Hypertension is responsible for 45% and 51% of deaths from heart diseases and strokes respectively.^5^ Hypertension scarcely exhibits symptoms in the early stages and many people go undiagnosed. In low- and middle-income countries where health systems are weak, the population is affected disproportionately.^5^ Although hypertension is not very prevalent in the low- and middle-income countries, the huge population of these countries gives rise to an increased number of people affected with hypertension. In 2008, the WHO reported that hypertension is most prevalent in the African region with about 46% of adults aged 25 years and above living with hypertension.^4^ In Ghana, the prevalence ranges between 25% and 29.4%.^6,7^

Diabetes mellitus is also known to exert a substantial burden in the sub-Saharan African region. People living with diabetes face significant challenges accessing diagnostic and treatment options. This contributes to increasing mortality and complications.^8^ However, a study conducted in Middlesbrough, UK, revealed a substantial inverse relationship of increasing prevalence of type 2 diabetes and low socio-economic status, which is markedly observed between the ages of 40 and 69 years.^9^

Accessibility and utilisation of healthcare services by patients with NCDs are largely influenced by some sociological factors. Studies conducted in the African setting suggest that patients living with NCDs are likely to seek healthcare from the formal health sector.^10^ Although not widely documented, some patients living with NCDs prefer the services of traditional medicines because of medical pluralism and western medicine being inefffective.^11,12^ The fact that patient needs, the available healthcare services, the social characteristics of individuals and the society have an immense influence on the individuals’ choice of facility is well documented.^13^ The burden of NCDs in Ghana is projected to increase because of ageing, rapid urbanisation and unhealthy lifestyles. It is therefore useful that some key factors such as prevalence of NCDs, the health-seeking behaviour (HSB) and factors influencing HSB are examined. There is an urgent need to develop efficient strategies that will halt the trend of rapidly growing morbidity and mortality and also effectively manage individuals diagnosed with NCDs.

## Objective

The aim of this study was to determine the prevalence of hypertension and diabetes in rural districts of Ashanti and identify factors that influence the choice of facility when seeking healthcare services. This information when available can be used readily to improve the management of hypertension and diabetes in the region and the healthcare system in general.

## Methods

### Study design

A population-based, prospective, cross-sectional study design was employed to assess the burden of hypertension and diabetes and to determine the factors that influence utilisation of health facilities by persons affected with hypertension and diabetes in rural districts of the Ashanti region, Ghana. The study was conducted between January 2016 and March 2016.

### Study area

Rural districts were defined as those that had more than 50% of their inhabitants in rural communities as indicated by the 2010 population census report of the Ashanti region of Ghana. The population size of the four districts selected were Offinso North (56 881), Amansie West (134 331), Ahafo Ano South (121 659) and Asante Akim South (117 245), with rural populations of more than 74%. The main occupation of the people in these districts was farming. The health facilities of the districts that constituted the study area are described in Table 1. On an average, a district has between 4 and 8 medical officers, 3–5 physician assistants, 2–3 pharmacists, 2–3 medical laboratory technicians, 80–120 nurses and few other supporting staff. The district hospital in a rural setting usually has an overall bed capacity of between 60 and 100. Some of the services offered include maternal and child health services (including immunisations), laboratory, pharmaceutical care services, voluntary counselling and testing (VCT) and antiretroviral therapy (ART) for HIV-infected patients, ear, nose and throat (ENT) services, nutrition and rehabilitating services, surgical services, out-patient and in-patient services in internal medicine among others. Each district also has between 8 and 12 health centres managed by physician assistants and nurses where patients are mainly seen on an out-patient basis, although patients can be admitted for not more than 24 h. Some health centres however have in addition a maternal health unit. The 10–15 community health planning services (CHPS) compounds in the district are manned mainly by community health nurses. In each district, there are private hospitals, traditional healers, an average of 12 over the counter medicine shops (OTCMS) can also be found, which are managed mainly by assistants present at the medicine counter. There are six pharmacies managed by pharmacists and are privately owned facilities.

**TABLE 1:** Health facilities at the study districts.

Health facility	Asante Akim South Municipal	Ahafo Ano South	Offinso North	Amansie West
Hospitals	2	1	1	2
Health centres	8	4	3	7
Community clinic	2	0	1	2
Maternity clinic	1	0	0	2
CHPS compound	5	8	1	11
Mission	0	3	2	0
Private	0	3	0	0

*Source:* Regional Health Directorate, Ashanti region.^15^

CHPS, Community Health Planning Services.

### Sample size estimation and sampling procedure

The sample size was calculated based on an estimated 50.9% prevalence of hypertension in rural districts in Ghana (1),^14^ a 95% CI which corresponds to 1.96 standard values, an alpha of 5%, a precision error of 4% and 15% non-response rate. The sample size was therefore estimated as 690.

A multistage sampling method was employed to enrol study participants from four rural districts (Amansie West and Offinso North, Asante Akim South and Ahafo Ano South) in the Ashanti region of Ghana. The districts were randomly selected based on the 2010 population census, and classification of a rural population taking the location of the district from the Metropolis (Kumasi) into consideration. Districts that shared borders with the Metropolis were not included in the list of districts from which the four districts were randomly selected. This was intended to minimise the possible influence of the urban population on the rural population. In the communities, four clusters were formed and an equal number of participants were enrolled from each cluster. The first household was conveniently sampled, after which every fourth house was sampled. About 700 prospective participants were contacted, 684 consented and 16 refused.

### Data collection and analysis

Data collection was conducted by the use of an electronic medium (Open Data Kit). Eight trained research assistants used computer tablets containing the preloaded questionnaire to collect demographic data, predisposing and enabling factors in relation to HSB of the respondents. Respondents also provided information on their preferred health facility when taken ill. Participants were also asked if they had been diagnosed with either hypertension or diabetes or both.

The data were exported to Stata version 13.0 (StataCorp., Lakeway Drive Station, TX, USA) for statistical analysis. Basic summary statistics of socio-demographic variables were conducted.

A wealth index was constructed for income and the socio-economic status index of the study respondents from household asset data by using principal components analysis^16^ and categorised as low, medium and high. The income status index was built by using three main income variables: the number of people who earn an income in the household, average monthly income of the household and an additional money support for the household. The socio-economic status index for this study was constructed by using 13 variables: an earned income, average monthly income, received additional support, completed senior secondary school, death of children aged less than 5, number of school-going children under the age of 5, number of rooms, type of materials used to make the wall of the house, house wired, have toilet facility, type of toilet facility, type of fuel and the number of meals served in a day in a household.

A chi-square test of association or a Fisher’s exact test was conducted to compare categorical variables and the outcome variable (HSB) as applicable. The HSB is a model of health services developed in the 1960s by Ronald M. Andersen (Andersen 1995; Petrovic & Blank 2015), and it is used to explain healthcare utilisation.^14,17,18^ The model has gone through a series of modifications and is used extensively in health service research in assessing utilisation. Finally, the multinomial logistic regression model was used to establish an association between HSB and predisposing, and enabling factors as proposed by Andersen’s behavioural model of health services. The multinomial logistic regression model is suitable for comparing more than two possible outcomes; it picks a base category and calculates the odds (relative risk ratio, RRR) of the other possible outcomes relative to it.

### Ethical consideration

This study involved human participants, hence permission to conduct the study (with the ethical clearance number: CHRPE/AP/503/17) was obtained from the Committee on Human Research Publications and Ethics, of Kwame Nkrumah University of Science and Technology. Participants were given detailed information regarding the purpose, the potential risks and benefits of the study. Voluntariness to participate was key in the process. Participants who agreed to participate signed the informed consent document to affirm their willingness to participate.

## Results

A total of 684 participants were recruited into the study (Table 2). The median age was 40 years with an interquartile range (IQR) of 28–55.50 years. About half of the participants (344; 50.29%) had attained basic education, although 167 (24.42%) had no formal education. Almost all participants perceived their current health status and that of the past year to be good. About 78% of participants (532; 77.78%) had registered with the National Health Insurance Scheme (NHIS). Table 3 shows the self-reported prevalence of hypertension and diabetes in the study population. The prevalence of hypertension was 111 (16.23%). Diabetes was prevalent in 37 (5.41%) of the study participants; thus, the prevalence of hypertension and diabetes was 137 (20.02%). The prevalence of diabetes and hypertension as a comorbidity was 11 (1.61%). About 60% of the participants with a history of hypertension and diabetes have lived with the disease for 1–5 years, with one person having lived with the disease for 11–15 years. Majority (115; 84.21%) of the participants came to know about their hypertension and diabetes upon visiting a hospital. Other means such as the pharmacy and community health screening were also mentioned. The main choice of facility preferred by patients identified with hypertension and diabetes included hospitals and health centres as mentioned by 72 (52.56%) and 55 (40.14%) respondents respectively. A little above half of the respondents (73; 53.28%) cited quality of care as the main reason for their preferred health facility followed by distance, which was also cited by 48 (35.04%) respondents (Table 3). The predisposing factors that could influence the preference of health facility for health seeking were age (*p* < 0.001), marital status (*p* < 0.001), educational level (*p* < 0.001) and occupation (*p* < 0.001) (Table 4). Preference for health facility was influenced by income status (*p* < 0.001) and socio-economic status (*p* < 0.001) (Table 5). Multinomial logistics regression model on the predisposing factors showed that educational level significantly decreased the likelihood of seeking healthcare in OTCMS and health centre when compared with hospitals (RRR = 0.1, 95% confidence interval [CI] = 0.011–0.917, *p* = 0.042 and RRR = 0.4, 95% CI = 0.198–0.679, *p* < 0.001) respectively (Table 6). Table 7 summarises factors that were not significant in seeking health care at a preferred facility in rural setting of Ashanti region (Table 7).

**TABLE 2:** Background characteristics of respondents.

Variable	Frequency (*n* = 684)	Percentage
**Sex**		
Male	356	52.05
Female	328	47.95
**Age years (mean [s.d.] = 43 ±17; median [IQR] = 40 [28.00–55.50])**		
< 26	112	16.37
26–35	171	25.00
36–45	117	17.11
46–55	113	16.52
56–65	99	14.47
> 65	72	10.53
**Marital status**		
Single	169	24.71
Married	389	56.87
Co-habiting	14	2.05
Separated	16	2.34
Divorced	23	3.36
Widowed	73	10.67
**Religion**		
Christian	544	79.53
Moslem	102	14.91
Traditional	23	3.36
Other	15	2.19
**Educational level**		
None	167	24.42
Basic level	344	50.29
Secondary level	116	16.96
Tertiary level	57	8.33
**Perceived present health status**		
Excellent	72	10.53
Very good	307	44.88
Good	254	37.13
Fair	40	5.85
Poor	11	1.61
**Perceived status over the past year**		
Excellent	42	6.14
Very good	311	45.47
Good	253	36.99
Fair	60	8.77
Poor	18	2.63
**Income status**		
Low	1029	60.42
High	674	39.58
**Socio-economic status**		
Low	426	25.01
Medium	426	25.01
High	426	25.01
Highest	425	24.96
**Enrolled on NHIS**		
Yes	532	77.78
None	152	22.12

s.d., standard deviation; IQR, interquartile range; NHIS, National Health Insurance Scheme.

**TABLE 3:** Prevalence of self-reported hypertension and diabetes.

Variable	Frequency (*n*)	Percentage
**Self-reported hypertension and diabetes status (*N* = 684)**		
Diabetes only	26	3.80
Hypertension and diabetes	11	1.61
Hypertension only	100	14.62
None	547	79.97
**History of disease condition (*n* = 137)**		
Less than a year	43	30.94
1–5 years	83	60.43
6–10 years	10	7.91
11–15 years	1	0.72
> 15 years	0	0.00
**Means of getting to know of status (*n* = 137)**		
Community health-screening programme	6	4.21
Church health-screening programme	13	9.47
Hospital	115	84.21
Pharmacy	3	2.11
**Preferred health facility (*n* = 137)**		
CHPS compound	0	0.00
OTCMS	5	3.65
Health centre	55	40.14
Hospital	72	52.56
Pharmacy	0	0.00
Private hospital	1	0.73
Traditional healer	0	0.00
**Reason for preference of a health facility (*n* = 137)**		
Available of medicine	5	3.65
Cost of care	1	0.73
Distance	48	35.04
Primary care provider (capitation)	10	7.30
Quality of care	73	53.28

CHPS, Community Health Planning Services; OTCMS, Over the Counter Medicine Shop.

**TABLE 4:** Chi-square test of independence of predisposing factors and preferred health facility for seeking healthcare in a rural setting.

Variable	Preferred health facility for seeking healthcare														*p*
	CHPS compound		OTCMS		Healthcare centre		Hospital		Pharmacy		Private hospital		Traditional healer		
	*n*	%	*n*	%	*n*	%	*n*	%	*n*	%	*n*	%	*n*	%	
**Age (years)**															< 0.001*
≤ 25	1	25.00	0	0.00	1	25.00	2	50.00	0	0.00	0	0.00	0	0.00	
26–35	0	0.00	0	0.00	2	66.67	1	33.33	0	0.00	0	0.00	0	0.00	
36–45	0	0.00	0	0.00	9	60.00	6	40.00	0	0.00	0	0.00	0	0.00	
46–55	0	0.00	1	2.63	14	36.84	23	60.53	0	0.00	0	0.00	0	0.00	
56–65	2	5.41	2	5.41	12	32.43	20	54.05	1	2.70	0	0.00	0	0.00	
> 65	1	2.50	2	50.00	17	42.50	20	50.00	0	0.00	0	0.00	0	0.00	
**Sex**															0.680*
Male	2	3.33	2	3.33	26	43.33	29	48.33	1	1.67	0	0.00	0	0.00	
Female	2	2.60	3	3.90	29	37.66	43	55.84	0	0.00	0	0.00	0	0.00	
**Marital status**															< 0.001*
Single	0	0.00	0	0.00	0	0.00	0	0.00	0	0.00	0	0.00	0	0.00	
Married	1	9.09	0	0.00	3	27.27	7	63.64	0	0.00	0	0.00	0	0.00	
Co-habiting	3	3.66	2	2.44	37	45.12	39	47.56	1	1.22	0	0.00	0	0.00	
Separated	0	0.00	2	66.67	1	33.33	0	0.00	0	0.00	0	0.00	0	0.00	
Divorced	0	0.00	0	0.00	2	50.00	2	50.00	0	0.00	0	0.00	0	0.00	
Widowed	0	0.00	1	2.70	12	32.43	24	64.86	0	0.00	0	0.00	0	0.00	
**Educational level**															< 0.001*
None	0	0.00	4	8.51	27	57.455	16	34.04	0	0.00	0	0.00	0	0.00	
Basic	4	5.80	1	1.45	22	31.88	41	59.42	0	0.00	1	1.45	0	0.00	
Secondary	0	0.00	0	0.00	5	29.41	12	70.59	0	0.00	0	0.00	0	0.00	
Tertiary	0	0.00	0	0.00	1	25.00	3	75.00	0	0.00	0	0.00	0	0.00	
**Occupational grouping**															< 0.001*
Farming	4	4.08	4	4.08	39	39.80	50	51.02	0	0.00	1	1.02	0	0.00	
Government worker	0	0.00	0	0.00	2	22.22	7	77.78	0	0.00	0	0.00	0	0.00	
Self-employed	0	0.00	0	0.00	8	47.06	9	52.94	0	0.00	0	0.00	0	0.00	
Student	0	0.00	0	0.00	0	0.00	1	100.00	0	0.00	0	0.00	0	0.00	
Unemployed	0	0.00	1	9.09	5	45.45	5	45.45	0	0.00	0	0.00	0	0.00	
Apprenticeship	0	0.00	0	0.00	1	100.00	0	0.00	0	0.00	0	0.00	0	0.00	

CHPS, Community Health Planning Services; OTCMS, Over the Counter Medicine Shop.

* , Fisher’s exact test was performed.

**TABLE 5:** Chi square test of independence of enabling factors and preferred health facility for seeking health in a rural setting.

Enabling factors	Preferred health facility for seeking healthcare														*p*
	CHPS compound		OTCMS		Healthcare centre		Hospital		Pharmacy		Private hospital		Traditional healer		
	*n*	%	*n*	%	*n*	%	*n*	%	*n*	%	*n*	%	*n*	%	
**Income status**															
Low	4	4.88	3	3.66	48	58.54	26	31.71	0	0.00	1	1.22	0	0.00	< 0.001*
High	0	0.00	2	3.64	7	12.73	46	83.64	0	0.00	0	0.00	0	0.00	
**Socio-economic status**															
Low	2	7.69	2	7.69	14	66.67	8	30.77	0	0.00	0	0.00	0	0.00	< 0.001*
Medium	0	0.00	1	3.85	11	42.31	13	50.00	0	0.00	1	3.85	0	0.00	
High	0	0.00	2	10.00	5	25.00	12	60.00	0	0.00	1	5.00	0	0.00	
Highest	0	0.00	0	0.00	0	0.00	37	100.0	0	0.00	0	0.00	0	0.00	0.00
**NHIS status**															
Yes	3	2.52	4	3.36	48	40.34	63	52.94	0	0.00	1	0.84	0	0.00	0.518*
No	1	6.25	1	6.25	6	37.50	8	50.00	0	0.00	0	0.00	0	0.00	

CHPS, Community Health Planning Services; OTCMS, Over the Counter Medicine Shop; NHIS, National Health Insurance Scheme.

* , Fisher’s exact test was performed.

**TABLE 6:** Multinomial logistics regression test of predisposing factors and preferred health facility for seeking health in the rural setting of Ashanti.

Preferred health facility/Hospital (base outcome)	RRR	*p*	95% CI
***CHPS compound***			
Age	0.9	0.837	0.378–2.200
Sex	0.8	0.824	0.086–7.047
Marital status	0.8	0.506	0.359–1.658
Education	0.8	0.823	0.183–3.861
Occupation	0.8	0.737	0.295–2.373
***OTCMS***			
Age	1.3	0.599	0.445–4.075
Sex	0.4	0.388	0.044–3.359
Marital status	0.8	0.503	0.455–1.471
Education	0.1	0.042	0.011–0.917
Occupation	0.7	0.286	0.297–1.431
***Health centre***			
Age	1.0	0.987	0.695–1.431
Sex	0.6	0.180	0.248–1.298
Marital status	0.8	0.096	0.625–1.039
Education	0.4	0.001	0.198–0.679
Occupation	0.9	0.476	0.596–1.273
***Private clinic***			
Age	2.6	0.433	0.234–29.657
Sex	-	-	-
Marital status	0.2	0.586	0.000–79.651
Education	0.7	0.790	0.050–9.769
Occupation	0.4	0.676	0.004–34.460

CHPS, Community Health Planning Services; OTCMS, over the counter medicine shop; RRR, relative risk ratio; CI, confidence interval.

**TABLE 7:** Multinomial logistics regression test of enabling factors and preferred health facility for seeking health in the rural setting of Ashanti.

Preferred health facility/Pharmacy (base outcome)	NCD		
	RRR	*p*	95% CI
**CHPS compound**			
Income status	-	-	-
Socio-economic status	1.5	0.803	0.052–45.344
NHIS status	1.9	0.708	0.064–57.524
**Health**			
Income status	0.4	0.284	0.052–2.372
Socio-economic status	1.8	0.649	0.153–20.285
NHIS status	0.6	0.656	0.052–6.445
**Hospital**			
Income status	2.3	0.392	0.340–15.664
Socio-economic status	13.1	0.095	0.639–267.263
NHIS status	0.6	0.684	0.055–6.704

NCD, non-communicable diseases; RRR, relative risk ratio; CI, confidence interval; CHPS, Community Health Planning Services; NHIS, National Health Insurance Scheme.

## Discussion

The prevalence of hypertension of 16.24% reported in this study is lower compared with previous studies conducted in Ghana with a prevalence of hypertension ranging from 25% to 35%.^7,19,20,21^ The study conducted by Agyemang, Bruijnzeels and Owusu-Dabo^19^ with a reported prevalence of 29.4% included subjects from both rural and urban districts in Ghana. This could be the reason for the decreased prevalence in this study. The prevalence was also found to be lower compared with a study conducted in Islamabad (Pakistan) for a semi-urban community with a reported prevalence of 38.7%.^22^ Some research findings however indicate that self-reported prevalence is usually underestimated for hypertension, although usually accurate for diabetes.^23,24^ The prevalence of hypertension could therefore be higher than stated.

The prevalence of diabetes of 5.41% found in the study population is consistent with a study conducted in a similar community in the Greater Accra Region of Ghana with a reported prevalence of 6.4%.^25^ The prevalence also reflects the general trend in sub-Saharan Africa where a systematic review indicated that the prevalence of diabetes ranges from 1% in rural Uganda to 12% in urban Kenya.^8^ The present trend of the prevalence of diabetes being similar and increasing in the study population is attributed to the ageing populations and increasing urbanisation of communities resulting in an additional burden of healthcare in developing countries where resources for managing clinical problems are usually scarce.^26^ Furthermore, the prevalence of diabetes in this study is much lower compared with a study conducted in southern India where the prevalence recorded was 18.6%.^27^

The burden of hypertension and diabetes is substantial in the study population; the rural communities in Ashanti Region have a fairly equal share of this public health challenge. The indication of the hospital as a preferred choice of health facility by about half of the participants with hypertension and diabetes indicates the growing acceptance of modern medicine in our part of the world. In Ghana and other sub-Saharan African countries, people with hypertension and diabetes are likely to prefer traditional medicine as opposed to modern medicine because of several factors.^11,12^ There was a statistically significant difference in their preference for the hospital among participants with hypertension and diabetes; the trend could be attributed to the fact that these are the available facilities that are also accessible to them. This finding is similar to a study conducted among women suffering from hypertension and diabetes who utilised public health facilities to manage their condition.^11^ The study results also compare with those of a study conducted in Esera, in Ethiopia, where the public health centre was the most preferred place for households seeking healthcare with a significant number of households preferring self-medication.^28^ However, the results differ from those from a study conducted in Bangladesh where about 70% of respondents preferred the pharmacy or chemical shops^29^ and also from those of a study conducted in Uganda where diabetic patients patronised traditional medicine.^12^

In a study conducted in South India to assess factors that influence care-seeking behaviour of participants with chest symptoms in both rural and urban communities, it was revealed that private healthcare facilities were the first and preferred choice of contact for about half of the rural participants. The major reasons included proximity to residence and their perception that good-quality care would be available.^30^ Similarly, the main reason for the choice of hospitals as a preferred health facility observed was perceived good quality of care offered. Proximity of the facility from their residence was also a reason for some participants; however, this was different from some research findings that indicate that cost of healthcare is the driving force for the choice of the health facility from which one seeks help.^31,32^ Again, the reason for choosing a facility in the rural setting reflects the findings of a study conducted in Mozambique where it was found that the proximity of the health facility increases the probability of seeking care in areas with poor resource.^33^ In our study, only 4 out of every 10 of the study participants in the study population cited the distance to the facility as the basis for their choice of health facility.

In this study, the predisposing factors (age, educational level attained, marital status and occupation) showed a trend of association between the HSB-preferred health facilities. A multinomial logistic regression model however showed no significant association between the predisposing factors and the choice of health facility. This finding indicates that choosing the most preferred facility depends on factors other than age, sex, marital status, educational level and occupation. This relates to a finding in Zambia where sex did not have a significant impact on one’s decision on where to seek healthcare.^34^ However, the study findings differ from a study that identified that education empowers people to include health-producing behaviour into rational lifestyle.^35^ The multinomial logistic regression model also revealed that in the rural setting, there was no significant association of enabling factors and participants suffering from hypertension and diabetes. Thus, the enabling factors (income and socio-economic status) had no influence on participants affected with hypertension and diabetes in their choice of a health facility.

## Limitations

Participants were classified as hypertensive and or diabetic based on self-reporting; this could result in a lower prevalence of the disease because some participants are usually in a state of denial when they are diagnosed with chronic diseases such as hypertension and diabetes. Furthermore, some participants might have the conditions but have not yet been diagnosed. Also, there could be a possibility of mismatch findings when comparing our results with previous studies that were conducted by employing the clinically diagnosed hypertension and diabetes methodology.

## Conclusion

Non-communicable diseases pose a great burden for the rural residents in the Ashanti region of Ghana. The prevalence of hypertension and diabetes observed in this study was 16.24% and 5.41% respectively. The hospital was identified as the most preferred choice among health facilities for patients suffering from hypertension and diabetes. The reason for their choice was the quality of services provided at the hospital. Educational level and enrolment in a health insurance scheme significantly increased the likelihood of seeking healthcare in public hospitals.
